# The “Magic Linker”: Highly Effective Gelation from Sterically Awkward Packing

**DOI:** 10.1021/acs.cgd.1c01470

**Published:** 2022-02-09

**Authors:** James P. Smith, Dmitry S. Yufit, James F. McCabe, Jonathan W. Steed

**Affiliations:** † Department of Chemistry, Durham University, Durham DH1 3LE, U.K.; ‡ Pharmaceutical Sciences, R&D, AstraZeneca, Macclesfield SK10 2NA, U.K.

## Abstract

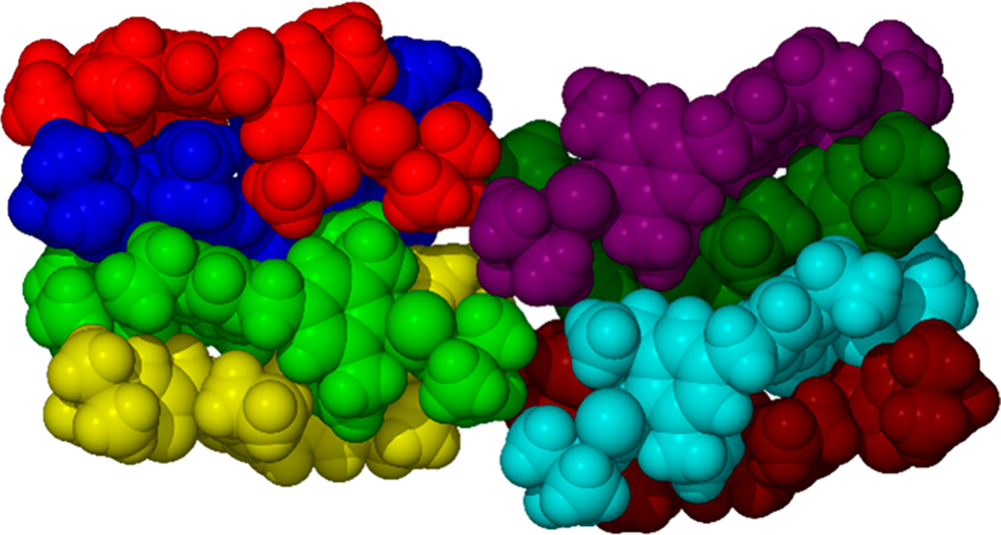

Bis(urea)s based on the 4,4′-methylenebis(2,6-diethylphenylene) (4,4′-MDEP) spacer are highly effective low molecular weight gelators, and the first single crystal structure of a bis(urea) based on this spacer is reported. The structure is a conformational isomorph with eight crystallographically independent molecules (*Z*′ = 8) arranged in four tennis-ball type dimers with the 2,6-diethylphenylene units adopting five different conformations in the ratio 4:5:3:2:2. The awkward shape and conformational promiscuity arising from the orientations of the ethyl groups in this system is linked to its gelation behavior. A total of seven 4,4′-MDEP derivatives have been prepared, and six are versatile gelators, confirming the particularly effective nature of the MDEP spacer. Only the nitrophenyl derivative does not form gels, likely because of intramolecular CH···O hydrogen bonding arising from the electron-withdrawing nature of the nitro substituent and hence inhibition of the urea α-tape hydrogen-bonded motif.

## Abstract 2

Gelators based on bis(urea) derivatives of 4,4′-methylenebis(2,6-diethylphenylene) are highly effective and versatile low molecule weight gelators. The first crystal structure of this class of compound proves to be a conformational isomorph with eight crystallographically independent molecules and five independent conformations. This conformational promiscuity offers an insight into the particularly effective gelation by this type of compound.

## Introduction

**Scheme 1:**
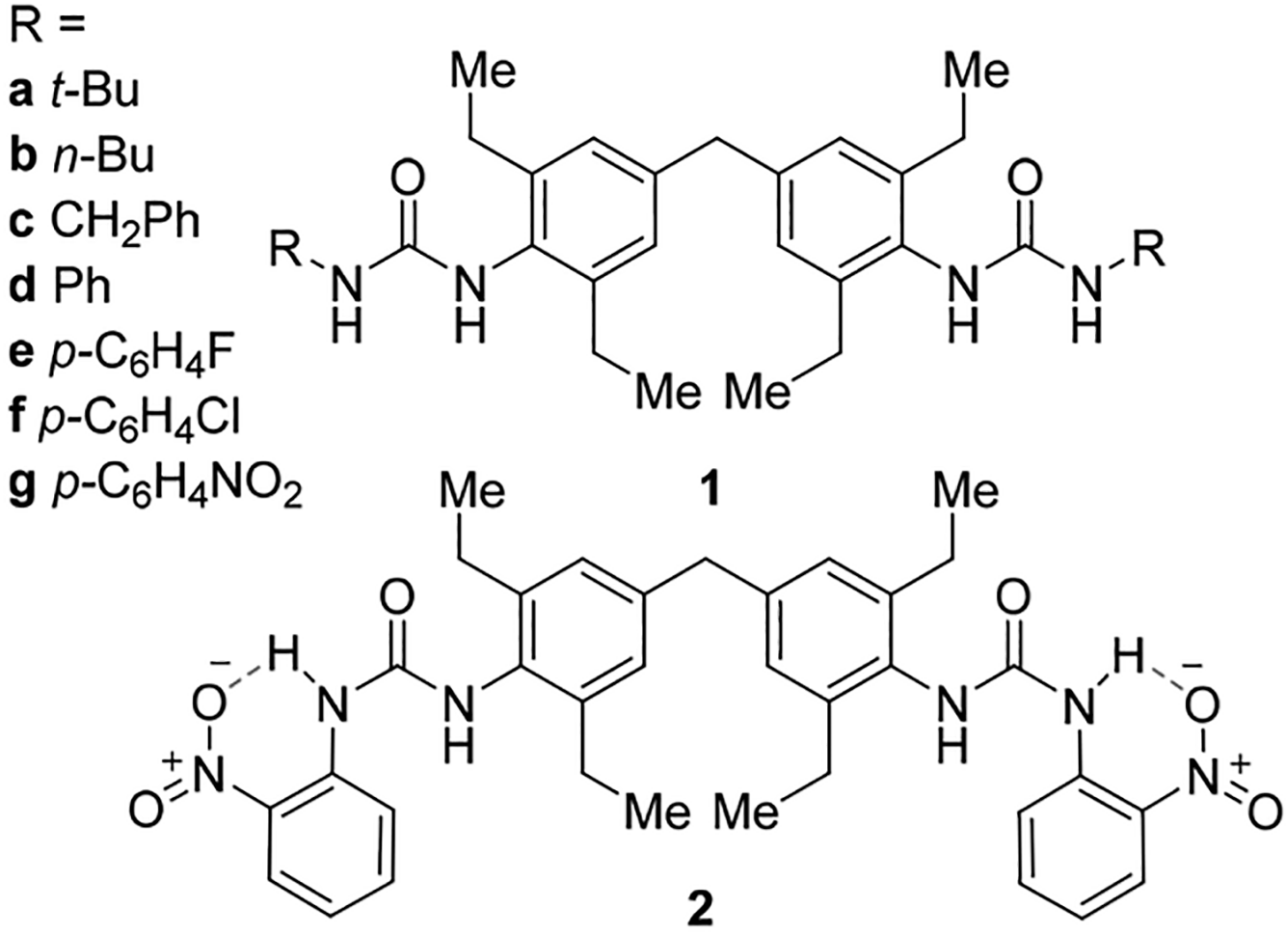
Molecular Structures of the Target Compounds **1a**–**1g** and the Related **2**, Which Adopts a *Syn–Anti* Urea Conformation Because of Intramolecular Hydrogen Bonding

Historically the discovery of low molecular weight gelators (LMWGs) has largely depended on serendipity, but recently LMWGs have been designed by incorporating functionalities known to promote supramolecular gelation and by modifying the structures of known LMWGs.^1,2^ Extensive work on bis(urea) LMWGs has shown that a simple structure incorporating a tunable spacer and peripheral substituents in addition to the urea hydrogen-bonding groups is a useful gel-forming template.^3−10^ LMWGs of this type are thought to give gelation by virtue of the common hydrogen-bonded urea α-tape motif.^3,4,11−13^ Gelation is tolerant of a range of peripheral substituents as long as the urea carbonyl acceptor group is not sterically hindered; however, it remains difficult to understand the effect of the spacer on gel formation. In extensive work on this class of compound, we have found that bis(urea)s based on the 4,4′-methylenebis(2,6-diethylphenylene) (4,4′-MDEP) spacer as in **1** tend to be highly effective gelators while closely related compounds with the same peripheral substituents but different spacers are often crystalline and either do not form gels or gel far fewer solvents.^7,14−16^ So effective is the 4,4-MDEP spacer that it is internally referred to as the “magic linker”. The steric bulk and hydrophobic nature of this spacer group are likely to be related to its tendency to form fibrous aggregates rather than crystalline materials; however, the details of the way in which assembles as part of bis(urea) gelators of type **1** remain unknown. While there is strong evidence the hydrogen-bonded urea α-tape synthon is commonly involved in the gelation behavior of this class of LMWG,^17^ crystal structure prediction (CSP) calculations on 4,4′-MDEP analogue **2** have shown that experimental XRPD patterns of the xerogels of this compound match computed crystal structures that exhibit an unusual eight-membered ring hydrogen-bonded synthon.^7^ However, compound **2** is atypical because of intramolecular hydrogen bonding to the nitro substituents (Scheme 1), and hence, experimental structural information on the aggregation of 4,4′-MDEP bis(urea) analogues would be extremely useful in explaining the particular effectiveness of this unit. Unfortunately, experimental crystal structure information on this class of compound is highly elusive specifically because of the tendency of these materials to form gels rather than single crystals. A CSD search reveals just one related single component structure of a nonethylated diphenylmethane quinolone bis(urea).^18^ While it is important to note that gel fibers are often polymorphic and molecular packing arrangements in crystal structures may not be representative of the packing in gel fibers,^19−23^ structural information on 4,4′-MDEP bis(urea)s would certainly provide valuable information regarding molecular conformations and packing tendencies that can potentially provide insight into the highly effective gelation behavior.^13,14,24^ In the present work, we focus specifically on bis(urea) derivatives of 4,4′-MDEP to probe the scope and tolerance of gels based on this spacer as a function of peripheral substituents and report the remarkable, “awkwardly packed” first single crystal structure of this class of compound as well as detailing the kind of approach necessary to crystallize these nearly uncrystallizable types of material.

## Results and Discussion

Bis(urea) 4,4′-MDEP analogues **1a**–**1g** were synthesized by reaction of 4,4′-methylenebis(2,6-diethylphenyl isocyanate) with the corresponding amines. The general synthetic procedure involves reacting the reagents under reflux in chloroform or THF, with a precipitate forming in most cases. This precipitate can be purified by washing with the reaction solvent and diethyl ether and then drying under vacuum at 110 °C. Synthetic and analytical details are outlined in the Supporting Information. The peripheral R-groups used were chosen to explore the influence of different structural features on the crystallization and/or gelation behavior of 4,4′-MDEP derived bis(urea)s, including comparisons between aromatic and aliphatic end groups and variation of steric bulk. The comparison of phenyl and benzyl end group in particular probes the influence of the accessibility of the urea carbonyl oxygen atoms since phenyl groups with electron-withdrawing substituents are expected to hinder the oxygen atom by formation of intramolecular CH···O hydrogen bonds,^6,25,26^ while the benzyl substituent allows unfettered access to hydrogen bond donors.^27^ The *tert*-butyl analogue **1a** was specifically included to generate steric hindrance near the urea functionalities by the bulky *tert*-butyl end groups. These steric effects may inhibit or slow down unidirectional urea hydrogen-bonded (likely α-tape type) assembly and allow crystallization rather than gelation and, hence, ideally, allow X-ray structural characterization of the compound.

### Gelation Behavior

**Figure 1:**
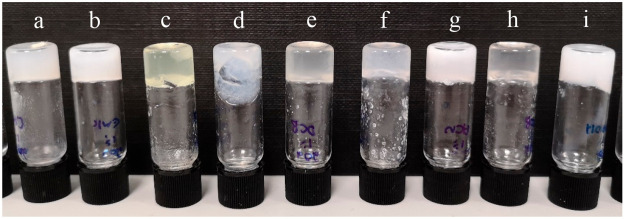
Gels of **1b** at 0.5% w/v in (a) chlorobenzene, (b) butan-2-one, (c) nitrobenzene, (d) toluene, (e) 1,2-dichlorobeznene, (f) 1,4-dioxane, (g) acetonitrile, (h) DMSO, and (i) ethanol.

The gelation behavior of compounds **1a–1g** was tested in a range of 16 solvents spanning the polarity spectrum, initially at 1% w/v on a 0.5 mL scale. Dissolution was aided by heating samples up to the boiling point of the solvent and then leaving the solution to cool to ambient temperature. After 24 h, samples were inverted to qualitatively determine gel-like behavior (Figure 1). If the solvent was completely immobilized, it was considered to have gelled. A partial gel was classified where only a fraction of the solvent was immobilized. The gelation results are shown below (Table 1).

**Table 1:**
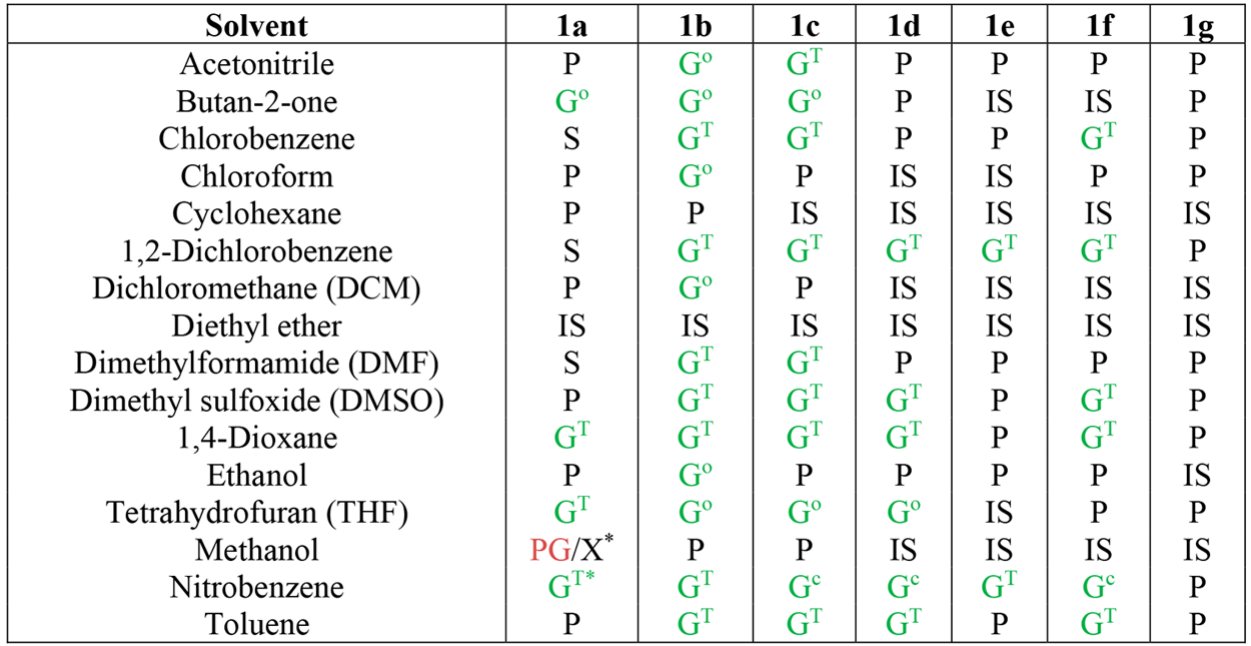
Gel Screen Results for Compounds **1a**–**1g** at 1% w/v in the Listed Solvents^a^

a G^T^ = translucent gel, G^c^ = clear/transparent gel, G^o^ = opaque gel, IS = insoluble, PG = partial gel, P = precipitate, S = solution, and X = crystallization. The asterisk indicates 2% w/v.

Analogue **1b** (R = *n*-butyl) is the most versatile gelator, gelling 13 of the 16 solvents tested, including nitrobenzene, 1,4-dioxane, DMF, and ethanol. The more sterically hindered *tert-*butyl derivative, **1a**, was far less versatile than **1b**, only gelling nitrobenzene at 2% w/v and THF, 1,4-dioxane, and butan-2-one at 1% w/v. Interestingly, **1a** also formed a partial gel in methanol at 1% w/v, but when the concentration was increased to 2% w/v homogeneous gelation did not take place, and instead very small crystalline needles forming concomitantly with a partial gel. This result supports the hypothesis that linear short alkyl chains are more versatile 4,4′-MDEP-derived gelators than branched alkyl groups and, hence the lack of steric hindrance near the urea carbonyl group is of importance in gelation.

Among gelators with aromatic peripheral groups, analogue **1c** (R = benzyl) is the most versatile LMWG, forming 10 gels, consistent with the unhindered nature of the substituent adjacent to the carbonyl oxygen acceptor. Analogues **1d** and **1f** each gel six solvents: nitrobenzene, 1,2-dichlorobenzene, 1,4-dioxane, DMSO, and toluene, with **1d** also gelling THF, while **1f** gels chlorobenzene. Compound **1e** (R = 4-fluorophenyl) is the least versatile gelator, only gelling nitrobenzene and 1,2-dichlorobenzene, while compound **1g** (R = 4-nitrophenyl) is a nongelator in all solvents tested. This evidence indicates that increasing the distance between urea functionality and aryl group improves the gel versatility, while electron-withdrawing para-substituents reduce the gelation capacity as a result of intramolecular CH···O hydrogen bonding to the urea carbonyl.^6,25,26^ This pattern of behavior points to the formation of the urea α-tape hydrogen-bonded motif being of importance in gelation in these systems.

### Rheological Properties

The 1,4-dioxane gels of compounds **1a**–**1d** and **1f** and the gel of **1e** in 1,2-dichlorobenzene were investigated using frequency and stress sweep rheometry measurements. All gels were at a concentration of 1% w/v and were formed directly on the rheometer plate. Gelation took 30–60 min to occur on the 2 mL scale, as opposed to a few minutes on the 0.5 mL scale, which is consistent with previous reports of how vial type and gel volume can influence gelation times and behavior.^28^

**Figure 2:**
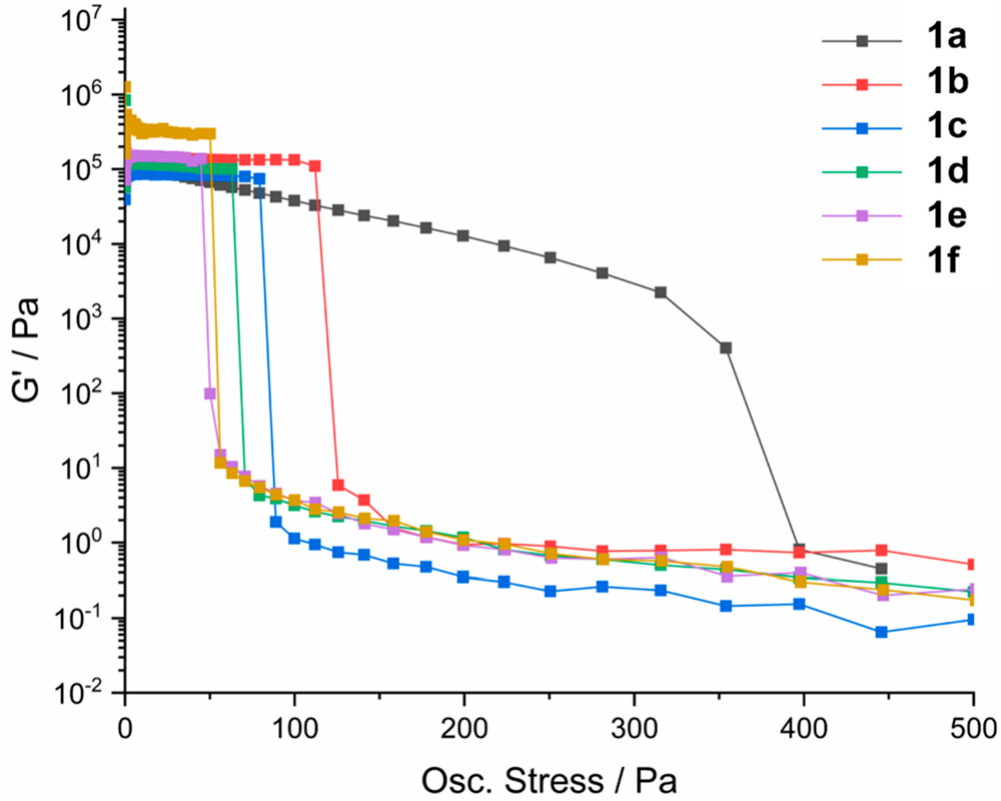
Oscillatory stress–sweep measurements for 1% w/v gels of **1a**–**1f**. The solvent was 1,4-dioxane for all gels, except **1e** where 1,2-dichlorobenzene was used. Measurements were taken at 10 °C to reduce solvent evaporation.

The frequency sweep measurements show that *G*′ > *G*″ by an order of magnitude for all samples, confirming their classifications as gels. All gels exhibit a *G*′ plateau of approximately 100 000 Pa. Oscillatory stress sweep measurements were performed over 0.05–1000 Pa with a constant frequency of 1 Hz (Figure 2). The gelators with aliphatic substituents **1a** and **1b** exhibit a higher yield stress than those with aromatic end groups. Interestingly, gels of **1a** (R = *tert*-butyl) exhibits a significantly higher yield stress than all the other gelators, which may reflect the tendency of this compound to crystallize. Gels of compound **1d** (R = phenyl) exhibit a lower yield stress than those of **1c** (R = benzyl) consistent with the less hindered acceptor, while **1e** (R = 4-fluorophenyl) and **1f** (R = 4-chlorophenyl) form weaker gels than **1d**, again consistent with the shielding of the urea carbonyl group by CH···O interactions. These measurements are consistent with *T*_gel_ measurements using the dropping ball method (Supporting Information, Table S1).

### Gel Morphology

**Figure 3:**
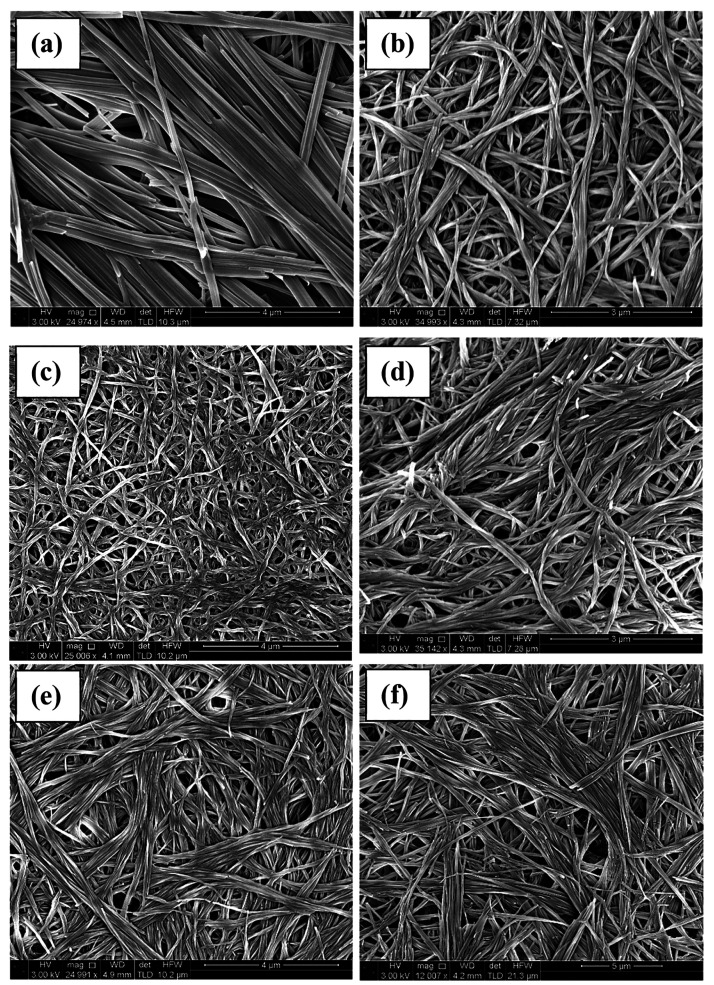
SEM images of xerogels of (a) **1a**, (b) **1b**, (c) **1c**, (d) **1d**, (e) **1e**, and (f) **1f**. All xerogels were formed from 1% w/v 1,4-dioxane gels, except for **1e**, which was formed from a 1% w/v 1,2-dichlorobenzene. Samples are coated with 2.5 nm platinum.

Small molecule supramolecular gels frequently comprise a fibrous network with a large surface area and high surface tension capable of immobilizing a fluid phase via capillary forces. However, the morphology of these fibers can be significantly different depending on such items as the LMWG molecular structure, the solvent immobilized, and the sample history.^29−31^ Careful drying of the gels can leave the fibrous network intact, allowing fibers to be imaged on the nanoscale using scanning electron microscopy (SEM). While this drying process can induce crystallization and changes in morphology particularly in gels of mixed solvents,^32^ this method frequently gives insight into the network morphology and microscale assembly. The 1,4-dioxane gels of compounds **1a**–**1d** and **1f** and a dichloroethane gel of **1e** were dried over 5 days at 50 °C, coated with 2.5 nm platinum, and imaged using SEM, Figure 3. Xerogel **1a** forms large, straight, long tape-like fibrils, approximately 500–800 nm in diameter with considerable parallel aggregation to form thicker fibers. The rather crystalline appearance of this material is consistent with the observed microcrystalline needles at high concentration from methanol. The other samples, **1b**–**1f**, are rather more curved and gel-like and quite similar to one another. The fibers entangle to form fibrous networks. Fiber diameters are typically 100–400 nm.

### Gelator Crystallization

**Figure 4:**
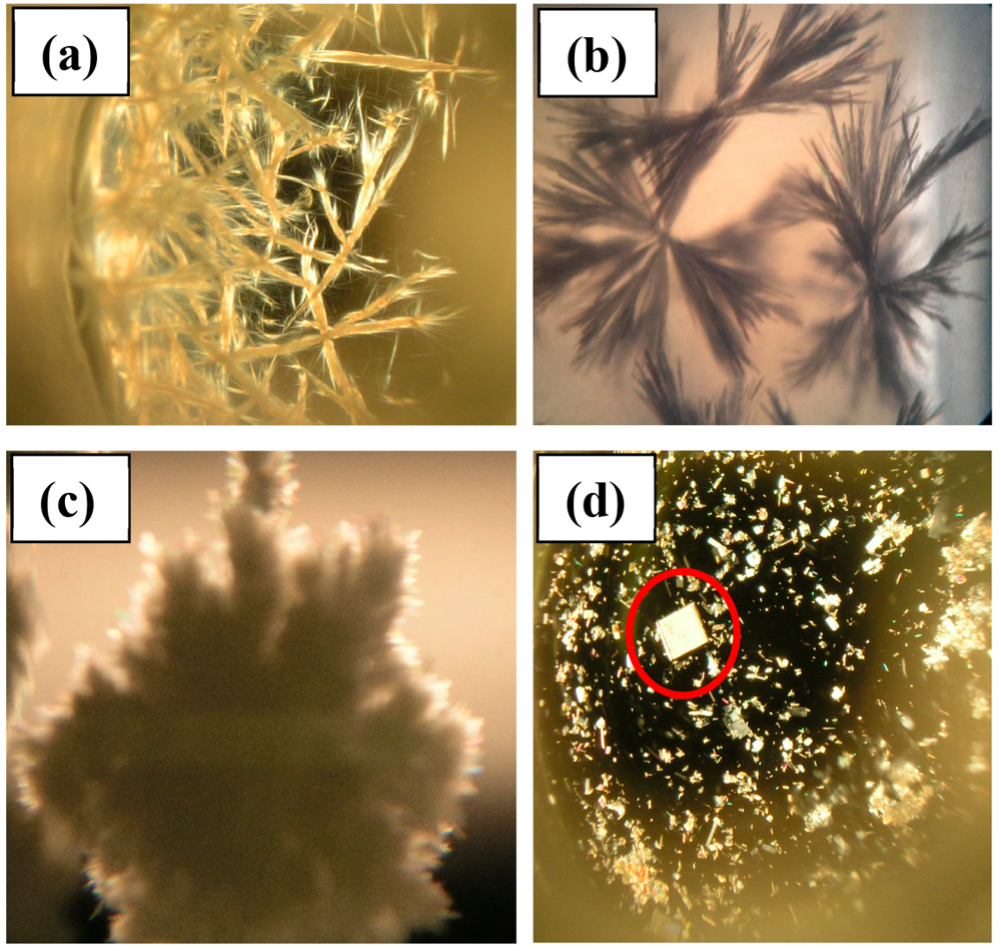
Optical microscopy images of semicrystalline or crystalline material obtained of (a) **1d** from 1,4-dioxane at 0.033% w/v, (b) **1e** from DMF at 0.05% w/v, and (c) **1c** grown from a 0.05% acetonitrile solution, slow cooled from 80 to 50 °C over 2 weeks. (d) The single crystal of **1a** (circled in red) grown from a 0.033% w/v THF solution via slow evaporation at ambient temperature for 2 days and then stored for a further 7 days undisturbed.

Crystallization of LMWGs is notoriously challenging due to the competitive nature of gelation and crystallization. Both gelation and crystallization occur under supersaturated conditions by aggregation of molecules but differ in the rate of particle growth along one direction. Hence it is unsurprising that growth of diffraction quality crystals with substantial size in all three dimensions for materials that, by definition, have a tendency toward one-dimensional aggregation is rarely achieved. Indeed, gelation may be considered as a kind of “frustrated” crystallization. Our interest in understanding the particular versatility of the 4,4′-MDEP motif led us to extensive efforts in order to crystallize gelators **1a**–**1f** with particular emphasis on the sterically hindered **1a**. The general crystallization strategy employed involved forming unsaturated solutions of gelators below the critical gel concentration and to steadily increase gelator concentration by either slow cooling of the solution or slow evaporation of the solvent (see Table S2 for conditions). This successfully yielded crystalline or semicrystalline material for **1a**, **1c**, **1d**, and **1e** (Figure 4). The remaining compounds either formed partial gels, stable solutions, oils, or amorphous powders. Attempts to optimize crystal growth conditions were made by lowering concentrations further and slowing the cooling or evaporation rate. The conditions used to grow the crystalline or semicrystalline material are detailed in the Supporting Information along with representative examples of unsuccessful crystallization conditions. Further optimization of the crystallization conditions for **1c**–**1e** failed to yield single crystals suitable for single crystal structure determination. While crystalline, these compounds exhibited a tendency to form very thin needles, aggregated in a dendritic morphology. Initially, similarly poor quality semicrystalline material of **1a** was obtained from THF solution. However, an iterative approach to adjusting the crystallization conditions to optimize crystal growth eventually yielded a single crystal of **1a** suitable for SC-XRD analysis. Details of the crystal growth procedure are given in the Supporting Information (Table S3) and involved progressive dilution to below the critical gelation concentration followed by slow evaporation until a single microcrystal was observed. The evaporation rate was then slowed considerably to optimize the growth of this particle while limiting nucleation of further microcrystals. The resulting sample is shown in Figure 4d.

The success in growing a diffraction quality sample of compound **1a** from a solvent in which it forms gels arises from a combination of this very careful crystal growth procedure and the deliberate design of this compound to sterically congest the urea carbonyl oxygen atom to slow the rate of hydrogen-bonded tape growth without completely turning off gelation.

**Figure 5:**
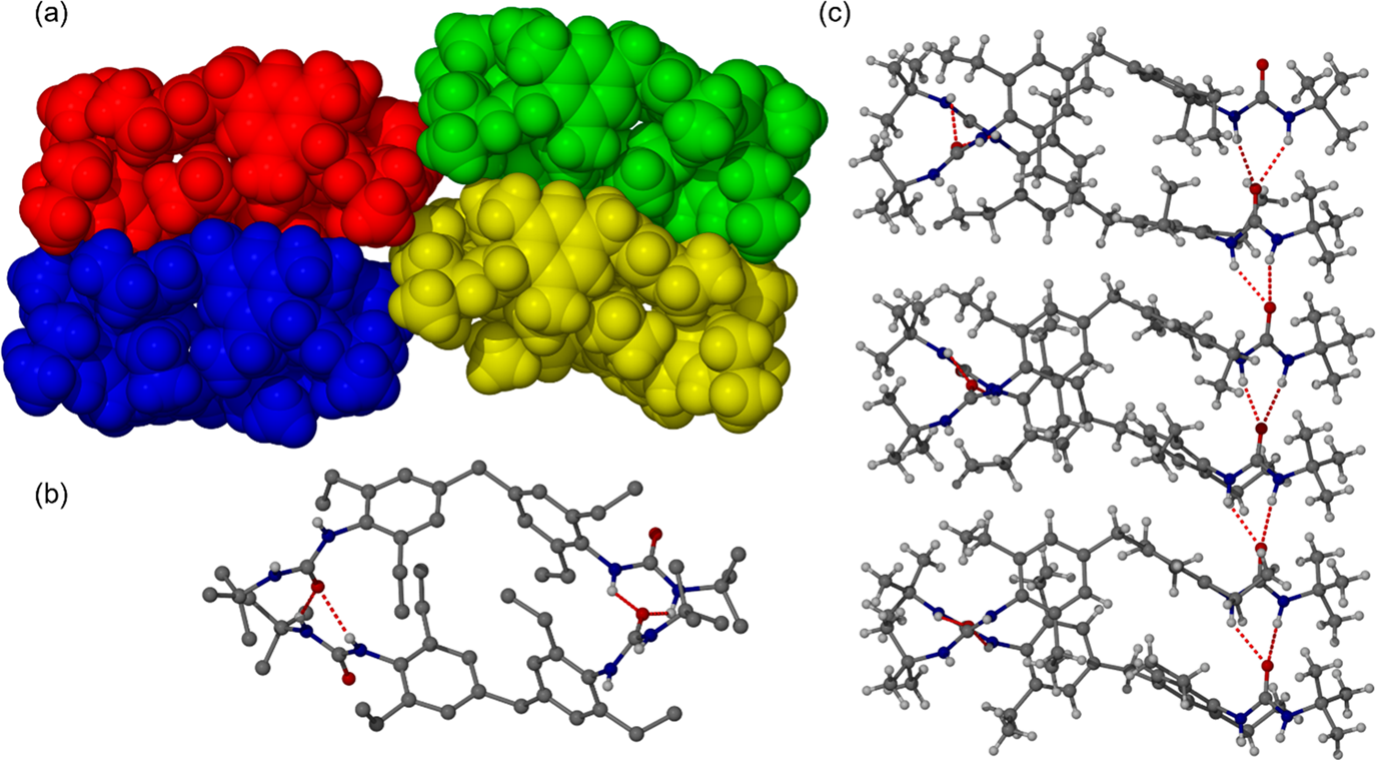
(a) Space filling view of the asymmetric unit of **1a** with each tennis ball like dimer colored differently. (b) Detail of one tennis ball shaped dimer showing the hydrogen-bonding arrangement. (c) Extended urea α-tape hydrogen-bonded chain.

The **1a** single crystal was analyzed by single crystal X-ray diffraction despite proving to be twinned. After the identification of a suitable twin law, the structure was successfully solved and refined to good precision despite the weakness of the diffraction (Table S4). The structure is remarkable in that it is a conformational isomorph^33,34^ with a total of eight crystallographically independent molecules (*Z*′ = 8), Figure 5a. Such a large number of symmetry-unique molecules is rare (and indeed other conformational isomorphs with high *Z*′ have been discredited^35,36^) and appears to arise from optimization of space filling by the adoption of multiple conformations for the ethyl substituents in conjunction with the formation of four tennis-ball like dimer motifs (Figure 5b) and two mutually orthogonal urea α-tape infinite hydrogen-bonded chains (Figure 5c). The dimeric motif allows the twisted 4,4′-MDEP core and the bulky *tert*-butyl motifs to effectively interdigitate in a chiral packing arrangement, Sohncke space group *P*2_1_. Frustration between multiple competing packing factors has been identified as a root cause of high *Z*′ behavior in a wide range of systems^37,38^ and seems to be of particular importance in the directional hydrogen bonding and awkward shape of this particular compound.

The structure shows that **1a** adopts an extended conformation with dihedral angles between the two aromatic rings close to 90° and near-coplanar C–CH_2_–C–CH torsion angles in the range 153–174°. This is a relatively unusual conformation for methylene-linked aryl groups, with only 479 structures out of 7688 reported in the CSD having a torsion angle greater than 160°. The conformation is likely adopted for derivatives of 4,4′-MDEP due to the steric hindrance generated by the ethyl substituents on each aryl group. This near-orthogonal dihedral angle between the aromatic rings results in each molecule forming two intermolecular *R*_2_^1^(6) urea α-tape synthons oriented at about 85° to one another. The N···O distances and N–H···O and N···C=O angles range between 2.8 and 2.9 Å, 146 and 150^o^, and 155 and 160°. These two orthogonal urea α-tape hydrogen-bonded motifs give rise to a lamellar structure with a 19.57 Å layer thickness corresponding to half the length of the crystallographic *c* axis. Lamellar packing in which opposite faces of the lamellae have very different surface energies (as may be expected in a chiral structure) have been shown to give rise to scrolling and hence gel fiber formation as opposed to flat layer-by-later packing to give 3D crystals.^17^

The CSP calculations previously undertaken on the 2-nitrophenyl 4,4′-MDEP analogue **2** revealed that the lowest energy conformers exhibit a folded conformation in the gas phase, with the end groups close together. However, higher energy extended conformers are stabilized in the solid-state to enable the formation of favorable intermolecular interactions such as intermolecular hydrogen bonding. The crystal structure of **1a** reveals an extended conformation with a conventional *anti–anti* orientation of the urea NH groups with respect to the urea carbonyl C=O bond^13,17^ and contrasts to the *syn–anti* conformation suggested for **2** by CSP calculations, which arises because of intramolecular hydrogen bonding to the nitro group.^7^ The perpendicular orientation observed in **1a** promotes self-assembly by hydrogen bonding in two dimensions, instead of the unidimensional assembly promoted by antiparallel urea tapes, and this may explain why **1a** is a less versatile gelator than the other 4,4′-MDEP analogues, and why the **1a** xerogel fibers are thicker and more ribbon-like than the fine cylindrical fibers observed for the other compounds.

**Figure 6:**
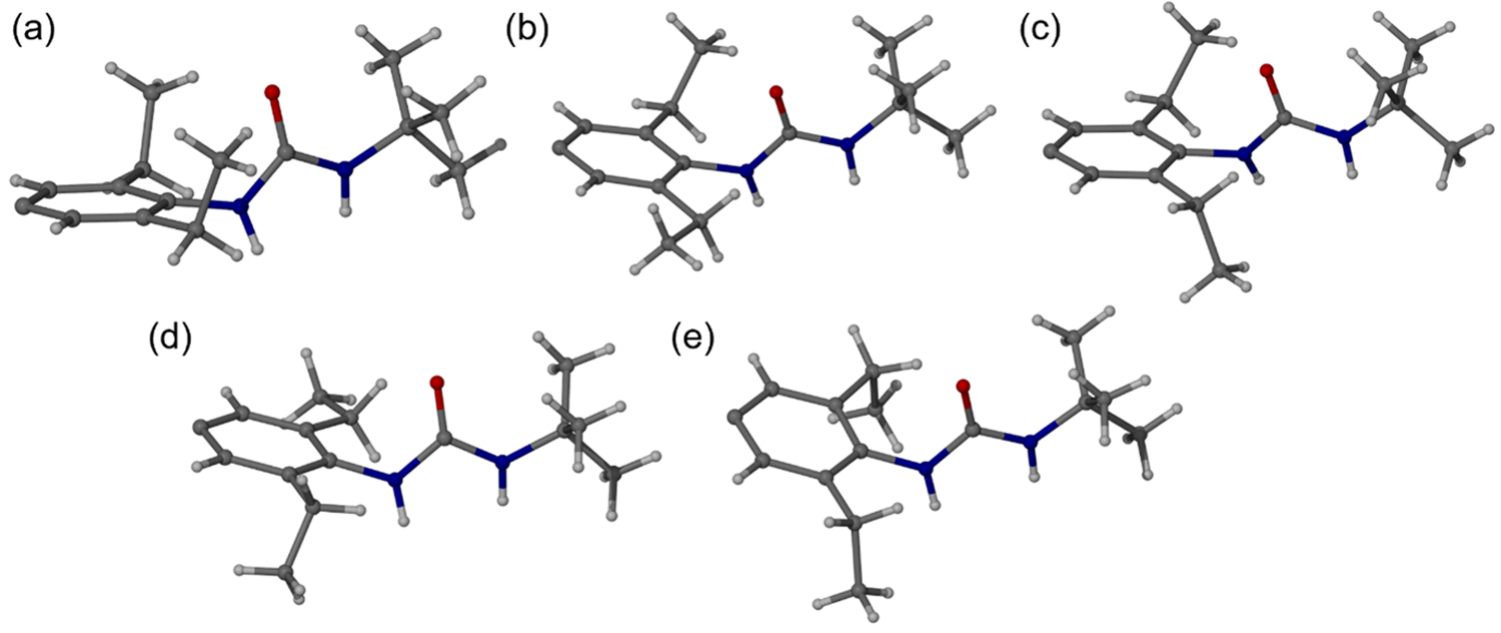
Distinct conformations of the ethyl groups on the 4,4-MDEP spacer observed in the **1a** crystal structure. (a) Axial_(up)_–axial_(up)_ where the ethyl CH_3_ groups are both oriented parallel to the urea carbonyl group above the plane of the aromatic ring. (b) Axial_(up)_–equatorial, where one CH_3_ is parallel to the urea carbonyl and one is parallel to the aromstic ring. (c) Axial_(up)_–axial_(down)_ where one ethyl CH_3_ group is oriented parallel to the urea carbonyl group above the plane of the aromatic ring while the other is in the opposite orientation below the ring plane. (d) Axial_(down)_–equatorial and (e) axial_(down)_–axial_(down)_.

The eight independent molecules differ most obviously in the orientation of the ethyl substituents, which are in principle free to rotate. Five distinct relative orientations of the ethyl groups can be observed in the structure (Figure 6). The ethyl substituents can be in an axial (up or down) conformation, where the CH_3_ group is above or below the plane of the aromatic ring respectively, or an equatorial conformation, where both CH_2_ and CH_3_ functionalities sit in the plane of the aromatic ring. The five conformations are axial_(up)_–axial_(up)_, axial_(up)_–equatorial, axial_(up)_–axial_(down)_, axial_(down)_–equatorial, and axial_(down)_–axial_(down)_. They are not all equally populated and occur in the ratio 4:5:3:2:2, respectively, hence precluding the description of the structure in a lower *Z*′ arrangement. This diversity may represent frozen out dynamic motion at the 120 K temperature of the structure determination or an optimization of space filling given the awkward shape of the twisted diphenylmethane-derived spacer.

**Figure 7:**
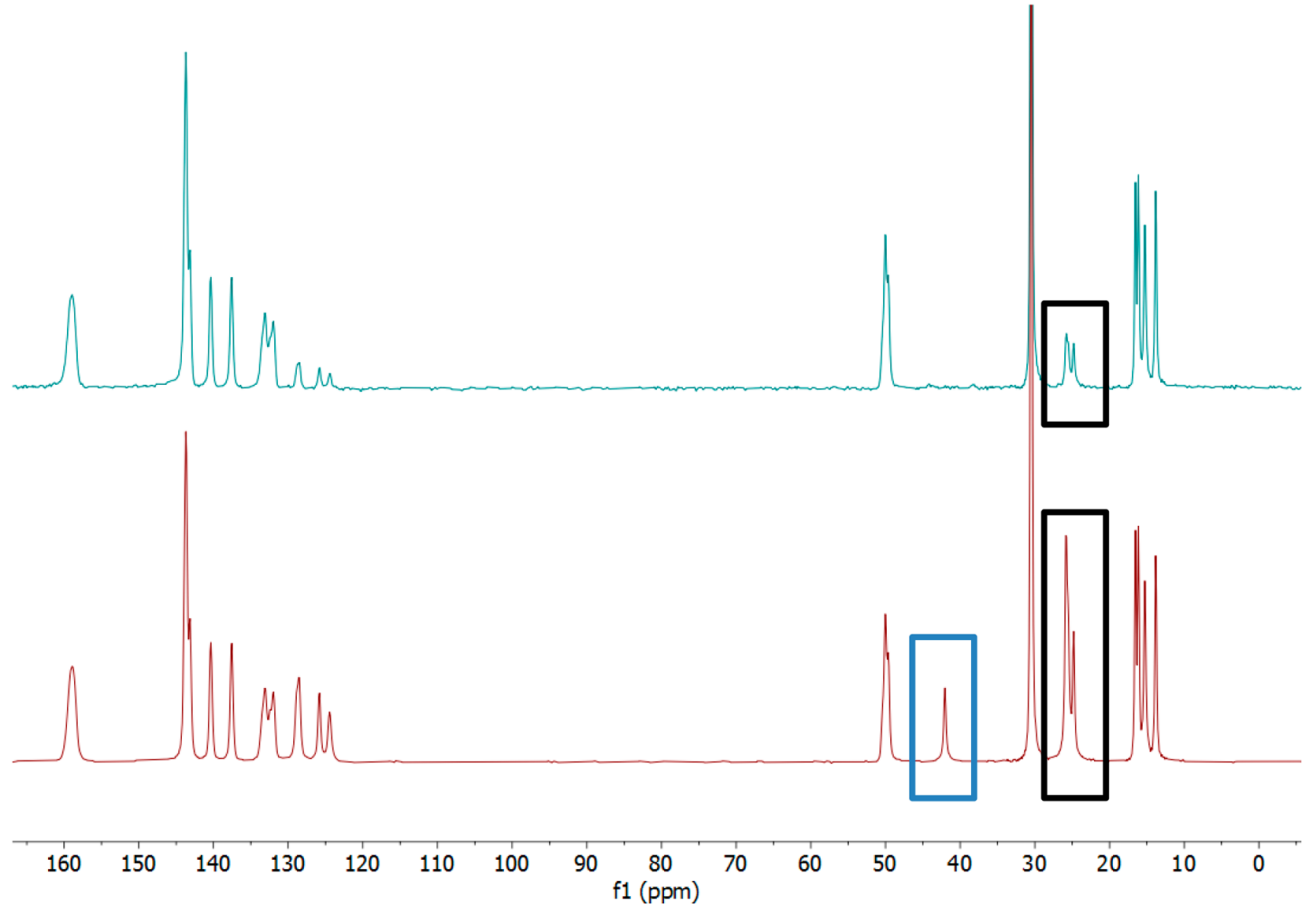
Solid-state ^13^C CP-MAS NMR spectra of **1a** with constant decoupling (bottom–in red) and interrupted decoupling (top–in green). The spectrum indicates at least four different environments for the methyl ends of the ethyl substituents consistent with the variety of environments observed crystallographically. The signal at 42.2 ppm (blue box) corresponds to the 4,4-MDEP spacer methylene unit and is fully suppressed in the interrupted decoupling spectrum, indicating the conformation is rigid. The signals at 25 ppm (black box) correspond to the ethyl *C*H_2_ and are partially suppressed in the interrupted decoupling experiment, indicating some degree of mobility at ambient temperature.

Seeding crystallizations were used to prepare approximately 100 mg of crystalline **1a** from methanol. The room temperature XRPD pattern of this bulk crystalline material matches closely the XRPD pattern of the dried xerogel. However, there are some differences between these room temperature patterns and the calculated pattern derived from the 120 K X-ray structure, although they appear to be the same phase. The experimental samples appear to be significantly affected by preferred orientation as a result of the anisotropic shape of the crystals, and differences arise from the change in unit cell size as a result of the temperature difference (Supporting Information, Figure S1). DSC measurements show no evidence for a phase change from −80 °C up to the 276 °C melting point of the sample (Supporting Information, Figure S2). The ^13^C CPMAS-NMR spectrum of crystalline **1a** at ambient temperature reveals sharp, well-defined signals showing high crystallinity. While the spectrum does not show direct evidence for the *Z*′ = 8 structure in the form of eight peaks for each carbon atom, there are four separate peaks assigned to the methyl termini of the ethyl substituents at around 15 ppm consistent with the different environments observed crystallographically (Figure 7). The aromatic region is similarly unsymmetrical, and resonances are somewhat broad. It is likely that the environments of the eight crystallographically independent molecules are sufficiently similar to one another that eight separate peaks for each carbon atom are not resolved. Instead, each *type* of ethyl environment gives rise to a separate resonance, giving four resonances in total between 10 and 20 ppm. The solid state ^13^C NMR experiment was repeated with interrupted decoupling, which suppresses signals from CH_2_ functionalities in a fully rigid system. This resulted in the complete suppression of the signal for the methylene CH_2_ group of the 4,4′-MDEP spacer at 42.2 ppm, indicating that the 4,4′-MDEP unit is rigid in the solid-state. The peaks at 24.9 and 25.9 ppm correspond to two environments for the CH_2_ functionalities of the ethyl groups decrease in intensity under interrupted decoupling, indicating restricted mobility even in these conformationally flexible groups under ambient conditions.

## Conclusions

As expected from previous studies the “magic-linker” 4,4′-MDEP proves to be a highly effective spacer in promoting gelation in bis(urea) LMWGs with every compound except for the nitroaryl derivative **1g**. The most effective gelators have the least steric hindrance about the urea carbonyl oxygen atom hydrogen bond acceptor. Careful control of supersaturation allows the crystallization of gelator **1a** and the determination of the single crystal structure of this effective bis(urea) gelator from a crystal grown from a gel medium. Such structures are extremely rare because of the tendency of gelators to form fibers. This is the first crystal structure determination of this linker design and reveals that the particular steric constraints of the 4,4′-MDEP spacer give rise to a tennis ball shaped dimer structure and with two mutually orthogonal urea α-tape hydrogen-bonded chains. The result is a chiral lamellar structure that is highly suited to the formation of 2D hydrogen-bonded sheets with very different surface energies with consequent scrolling of the lamellae to give twisted fibrils in accordance with our previously published model of gel formation in these types of system.^17^ The specific steric constraints of the 4,4′-MDEP spacer give rise to a remarkable *Z*′ = 8 chiral packing arrangement, with the ethyl groups adopting a variety of different conformations to fill space. This unsymmetrical arrangement in the solid state is clearly evident by the observation of four CH_3_ resonances in the solid state ^13^C NMR spectrum of the material. The crystalline structure appears to be representative of the gel phase although a phase transformation between gel and crystal cannot be ruled out. The awkward shape and tendency to adopt an interdigitated dimeric packing of the hydrophobic core in 4,4′-MDEP promote chiral 2D lamellar packing, and hence, this linker tends to promote gelation in every derivative as long as the 2D hydrogen-bonded sheet is not prevented by the inaccessibility of the urea carbonyl group.

## Supporting Information Available

Experimental details of the synthesis of compounds **1a**–**1g** and DSC and XRPD data as well as details of crystallization procedures and crystallographic experimental information (PDF)

## Accession Codes

CCDC 2124557 contains the supplementary crystallographic data for this paper. These data can be obtained free of charge via www.ccdc.cam.ac.uk/data_request/cif, or by emailing data_request@ccdc.cam.ac.uk, or by contacting The Cambridge Crystallographic Data Centre, 12 Union Road, Cambridge CB2 1EZ, UK; fax: +44 1223 336033.
