# Supplementary material for: The “Magic Linker”: Highly Effective Gelation from Sterically Awkward Packing
Source: Cryst Growth Des. 2022 Feb 9;22(3):1914–21. doi: 10.1021/acs.cgd.1c01470 (PMC9084547; doi:10.1021/acs.cgd.1c01470)
Supplement: Supplementary file 1 — cg1c01470_si_001.pdf [file cg1c01470_si_001.pdf]

The ‘Magic Linker’: Highly Effective Gelation from Sterically Awkward Packing

James P. Smith[a], Dmitry S. Yufit[a], James F. McCabe[b] and Jonathan W. Steed[a].*

(a) Department of Chemistry, Durham University, Durham DH1 3LE, UK. (b) Pharmaceutical Sciences, R&D, AstraZeneca, Macclesfield, UK.

Supplementary Information

Table S1. The results of ball-experiments undertaken to determine *T*gel (˚C). Ball-drops were undertaken on a 2 mL scale using a ball bearing. NG = no gel in this solvent.

Solvent	**1a**	**1b**	**1c**	**1d**	**1e**	**1f**
1,4-dioxane	83	67	58	50	NG	41
1,2-dichlorobenzene	NG	112	108	95	88	90

Table S2. The conditions used to grow crystalline material of **1c-e** pictured in Figure 4. Outcome abbreviations: X = crystalline or semi-crystalline aggregates unsuitable for SC-XRD analysis.

**Compound**	**Solvent**	**Procedure**	**Outcome**
**1c**	Acetonitrile	2 mg **1c** was dissolved in 4 mL acetonitrile with heat. The vial was covered with pinpricked parafilm so solvent could slowly evaporate. After 5 days, aggregates of microcrystalline needles could be observed by eye.	X

**1d**	1,4-dioxane	2 mg **1d** was dissolved in 6 mL 1,4-dioxane with heat. The resulting solution was cooled from 98 ^{o}C to 50 ^{o}C over 2 weeks. Within this time, bundles of entangled semi-crystalline fibres formed.	X
**1e**	DMF	4 mg **1e** was dissolved in 8 mL DMF with heat and sonication. The solution was slowly cooled from 140 ^{o}C to 50 ^{o}C over 4 weeks. This yielded small, poor quality crystalline needles that were highly twinned and not suitable for SC-XRD.	X

Table S3. The method development used to grow a single crystal of **1a**. The first sentence (in bold) in each crystallisation procedure states how the method in each iteration differs from the previous. Outcomes: PG = partial gel, S = stable solution, X = crystalline powder, SC = single crystal.

**Iteration**	**Crystallisation Procedure**	**Outcome**
1	Compound **1a** (10 mg) was dissolved in THF (3 mL) with heat. The resulting sol was allowed to cool to ambient temperature. After 12 hours, the solvent was partially immobilised.	PG
2	**The concentration was decreased by half.** Compound **1a** (5 mg) was dissolved in THF (3 mL) with heat. The resulting sol was allowed to cool to ambient temperature. After 12 hours, a mixture of partial gel and small crystalline precipitate was observed.	PG and X
3	**The concentration was decreased significantly further.** Compound **1a** (2 mg) was dissolved in THF (7 mL). The resulting sol was allowed to cool to ambient temperature. After 1 week, the solution was still stable with no precipitate or gel observed.	S
	**The concentration of the sol formed in iteration 3 was increased by slow**	

4	**evaporation.** The vial cap for the sol in iteration 3 was loosened and left undisturbed for 1 week. After this, around 2 mL of THF was left in the vial and a crystalline precipitate had formed.	X
5	**The rate of solvent evaporation was reduced.** Compound 1**a** (2 mg) was dissolved in THF (7 mL). The resulting sol was allowed to cool to ambient temperature. The vial was then covered with parafilm instead of a cap. A single pinprick was put in the parafilm to allow solvent to slowly evaporate. After 5 days, approximately 2 mL THF had evaporate and a couple of small crystals just identifiable by eye had formed. The vial was left untouched for a further 2 days with solvent evaporation still enabled, during which time, mass nucleation occurred, and a lot of small poor-quality crystals were obtained.	X
6	**Solvent evaporation was restricted once the first crystalline seeds were observed by eye.** The rate of evaporation was reduced. Compound **1a** (2 mg) was dissolved in THF (7 mL). The resulting sol was allowed to cool to ambient temperature. The vial was then covered with parafilm. A single pinprick was put in the parafilm to allow solvent to slowly evaporate. After 5 days, approximately 2 mL THF had evaporate and a single small crystal just identifiable by eye had formed. The vial was then sealed with a cap, taking care to minimise movement of the vial. The vial was then left for a further 7 days, in which time the single crystal had grown to a sufficient size for SC-XRD analysis. Additional small crystals had also formed in this time but had not disrupted the growth of the major single crystal.	SC

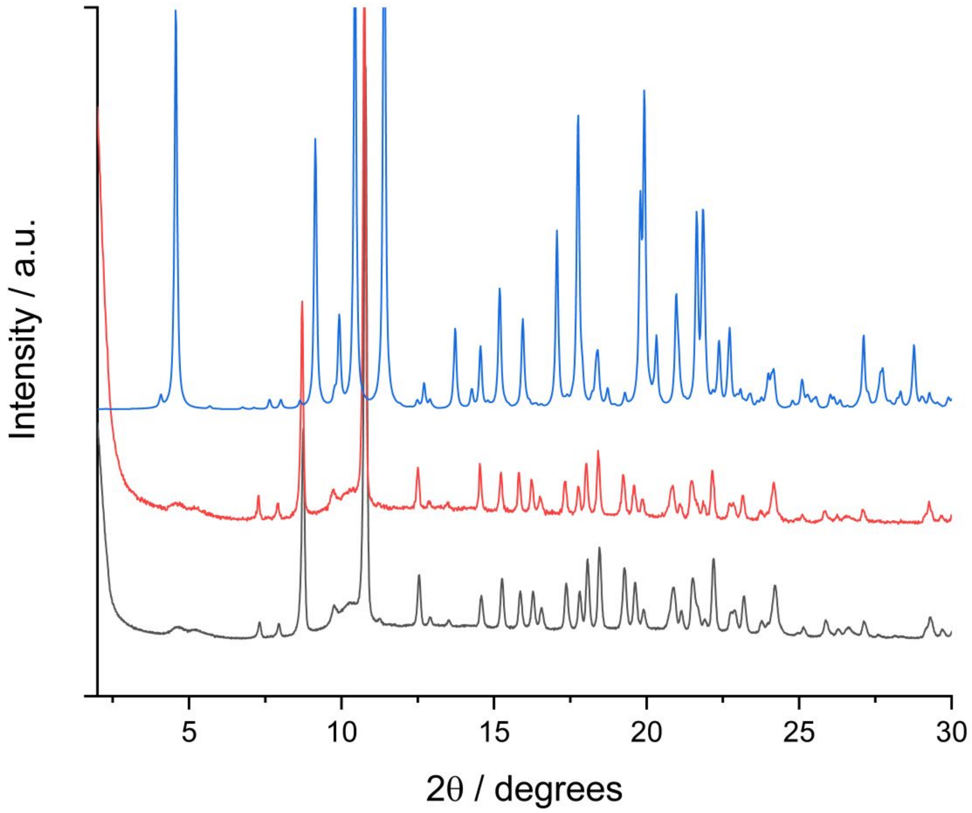

Figure S1. XRPD pattern calculated from the single crystal structure (in blue) and the experimental XRPD patterns for the bulk crystalline material obtained from seeding crystallisation (in red) and the **1a** 1,4-dioxane xerogel (in black). The xerogel and experimental crystal are closely correlated, although the appearance and high crystallinity of the xerogel specimen may indicate crystallisation has occurred upon drying and this pattern may not accurately represent packing in the gel phase. Both experimental patterns have similarities with the calculated pattern, although the difference in temperature and preferred orientation make comparison difficult. In particular the peak at 4.57^{o} is the (002) reflection and corresponds to the lamellar spacing of 19.57 Å. This peak appears to be greatly reduced in

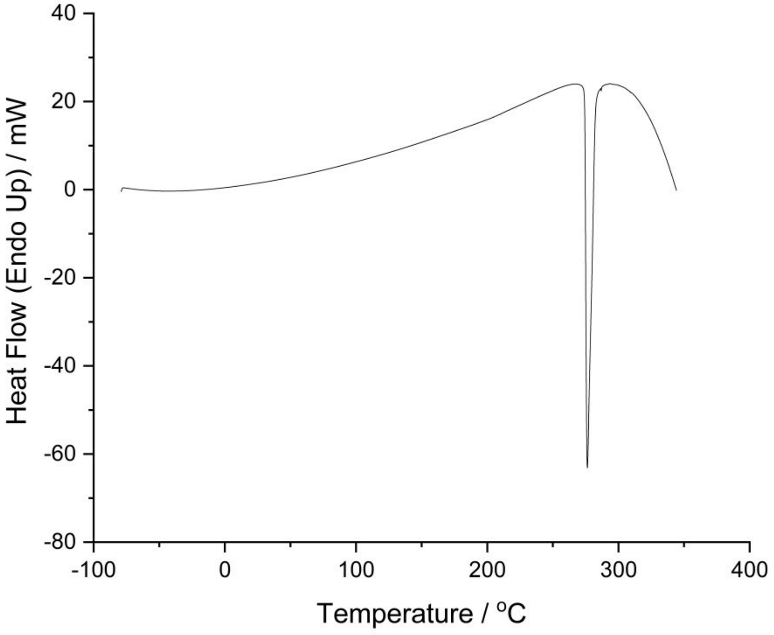

Figure S2. DSC thermogram of **1a**, showing no polymorphic phase transition between -80 °C and 350 °C, and a single melt endotherm at 276 °C.

General Synthesis Procedure

All bis(urea) compounds were synthesized by similar synthetic procedures. TetraethyI-4,4'-methylenediphenyl isocyanate (0.500 g, 1.380 mmol) was dissolved in a vial with chloroform (7 mL) or THF (7 mL) at ambient temperature with stirring. This was followed by the addition of 2.1 molar equivalents (2.90 mmol) of the desired primary amine. Details of the amine used in synthesis of each compound are provided for each compound below. For compounds **1a–1c**, the reaction mixture was stirred at room temperature for 2 to 4 hours, with white precipitates formed within minutes of stirring. For compounds **1d-g**, triethylamine (314 mg, 3.10 mmol) was also added to the reaction mixture before heating under reflux for up to 12 hours. Precipitates typically formed within 2 hours of reflux. For all compounds, the reaction mixture was filtered to obtain the crude precipitate before further purification steps.

Purification of compounds **1a–f** was achieved by washing the precipitate with dichloromethane (3 x 10 mL) and drying under vacuum in a drying pistol at 110 °C. Purification of the **1g** was achieved by triturating the precipitate with 15 mL DMF for 12 hours. The precipitate was then filtered and washed with copious amounts of water (150 mL), followed by washing with diethyl ether (3 x 10 mL). The solid was then dried under vacuum in a drying pistol at 110 °C for 24 hours.

Compound 1a

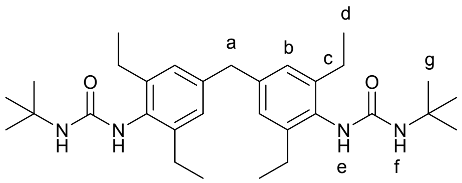

Primary amine: tert-butylamine; *Solvent:* THF

Compound **1a** was obtained as a white solid (599.89 mg, 1.18 mmol, 88.7 % yield). **^{1}H NMR** (400 MHz, DMSO-[d6]): δ = 7.09 (s, 2H, *e*) 6.91 (s, 4H, *b*), 4.50 (s, 2H, *f*) 3.80 (s, 2H, *a*). 2.49-2.55 (m, *c* – overlaps with DMSO peak), 1.26 (s, 18H, *g*) 1.07 (t, 12H, J = 7.6 Hz, *d*). **m/z (ES$^+$-MS)** 509.4 [M+H], 531.4 [M+Na]. **Elemental analysis** calculated for $C_{31}H_{48}N_4O_2$ (%): C, 73.19; H, 9.51; N, 11.01. Found (%): C, 72.93; H, 9.38; N, 10.76.

Compound 1b

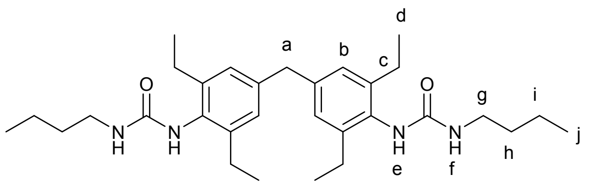

Primary amine: n-butylamine; *Solvent:* THF

Compound **1b** was obtained as a white solid (638 mg, 1.26 mmol, 91.3 %). **^{1}H NMR** (400 MHz, DMSO-[d6]): δ = 7.23 (broad-s, 2H, *e*) 6.87 (s, 4H, *b*), 5.33 (t, *J* = 5.7 Hz, 2H, *f*), 3.69 (s, 2H, *a*), 2.92 (dt, J = 6.3, 5.7 Hz, 4H, *g*), 2.49-2.55 (m, *c* – overlaps with DMSO peak), 1.38-1.18 (m, 8H, *h i*) 1.07 (t, 12H, J = 7.6 Hz, *d*), 0.91-0.73 (m, 6H, *j*). **m/z (ES$^+$-MS)** 509.4 [M+H], 531.4 [M+Na]. **Elemental analysis** calculated for $C_{31}H_{48}N_4O_2$ (%): C, 73.19; H, 9.51; N, 11.01. Found (%): C, 72.89; H, 9.26; N, 10.81.

Compound 1c

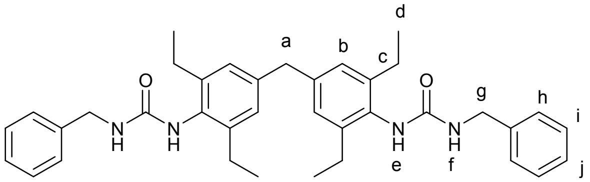

Primary amine: Benzylamine; *Solvent:* Chloroform

Compound **1c** was obtained as a white solid (669 mg, 1.16 mmol, 84 % yield). **^{1}H NMR** (400 MHz, DMSO-[d6]): δ = 7.40 (s, 2H,*e*) 7.22-7.41 (m, 10H, *h i j*), 6.95 (s, 4H, *b*), 6.88 (s, 2H, *f*), 4.25 (d, 4H, J = 5.8 Hz, *g*), 3.80 (s, 2H, *a*). 2.49-2.55 (m, *c* – overlaps with DMSO peak), 1.12 (t, 12H, J = 7.6 Hz, *d*). **m/z (ES$^+$-MS)** 577.4 [M+H], 599.3 [M+Na]. **Elemental analysis** calculated for $C_{37}H_{44}N_4O_2$ (%): C, 77.05; H, 7.69; N, 9.71. Found (%): C, 77.05; H, 7.67; N, 9.66.

Compound 1d

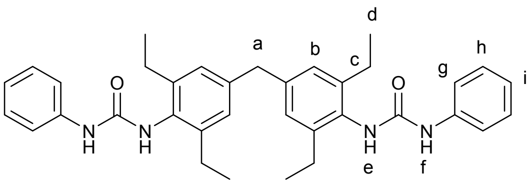

Primary amine: Aniline; *Solvent:* Chloroform

Compound **1d** was obtained as a white solid (537 mg, 0.980 mmol, 71 % yield). **^{1}H NMR** (400 MHz, DMSO-[d6]): δ = 8.74 (broad-s, 2H, *f*) 7.56 (s, 2H, *e*), 7.44 (d, 4H, J=7.7 Hz, *g*), 7.25 (m, 4H, *h*), 7.01 (s, 4H, *b*), 6.92 (m, 2H, *i*), 3.86 (s, 2H, *a*). 2.49-2.55 (m, *c* – overlaps with DMSO peak), 1.12 (t, 12H, J = 7.5 Hz, *d*). **m/z (ES^{+}-MS)** 571.3 [M+Na], 587.3 [M+K]. **Elemental analysis** calculated for $C_{35}H_{40}N_4O_2$ (%): C, 76.61; H, 7.35; N, 10.21. Found (%): C, 76.56; H, 7.38; N, 10.20.

Compound 1e

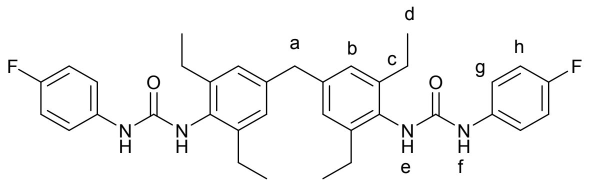

Primary amine: 4-fluoroaniline; *Solvent:* Chloroform

Compound **1e** was obtained as a white solid (524 mg, 0.897 mmol, 65 % yield). **^{1}H NMR** (400 MHz, DMSO-[d6]): δ = 8.76 (broad-s, 2H, *f*) 7.54 (s, 2H, *e*), 7.43 (d, J = 8.4 Hz, 4H, *h*), 7.29 (d, 4H, J = 8.4 Hz, *g*), 6.99 (s, 4H, *b*), 3.85 (s, 2H, *a*). 2.49-2.55 (m, *c* – overlaps with DMSO peak), 1.10 (t, 12H, J = 7.6 Hz, *d*). **m/z (ES^{+}-MS)** 585.3 [M+H], 607.3 [M+Na]. **Elemental analysis** calculated for $C_{35}H_{38}N_4O_2F_2$ (%): C, 71.90; H, 6.55; N, 9.58. Found (%): C, 71.81; H, 6.71; N, 9.64.

Compound 1f

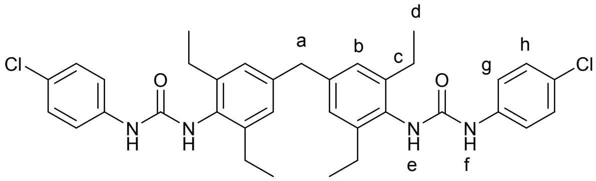

Primary amine: 4-chloroaniline; *Solvent:* Chloroform

Compound **2f** was obtained as a white solid (639 mg, 1.035 mmol, 75 % yield). **^{1}H NMR** (400 MHz, DMSO-[d6]): δ = 8.89 (broad-s, 2H, *f*) 7.61 (s, 2H, *e*), 7.46 (d, 4H, J = 8.8 Hz, *h*), 7.28 (d, 4H, J = 8.8 Hz, *g*), 7.00 (s, 4H, *b*), 3.86 (s, 2H, *a*). 2.49-2.55 (m, *c* – overlaps with DMSO peak),

1.11 (t, 12H, J = 7.6 Hz, *d*). **m/z (ES$^+$-MS)** 617.2 [M+H], 639.2 [M+Na]. **Elemental analysis** calculated for $C_{35}H_{38}N_4O_2Cl_2$ (%): C, 68.07; H, 6.20; N, 9.07. Found (%): C, 67.83; H, 6.35; N, 9.13.

Compound 1g

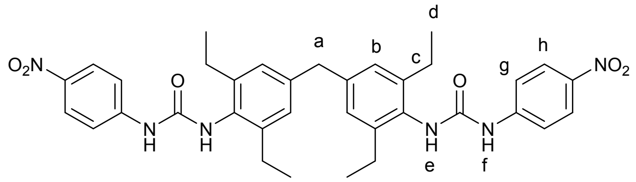

Primary amine: 4-nitroaniline; *Solvent:* Chloroform

Compound **1g** was obtained as a white solid (520 mg, 0.814 mmol, 59 % yield). ^{1}**H NMR** (400 MHz, DMSO-[d6]): δ = 9.58 (broad-s, 2H, *f*) 8.17 (d, 4H, J = 9.3 Hz, *h*), 7.86 (broad-s, 2H, *e*), 7.67 (d, 4H, J = 9.3 Hz, *g*), 7.03 (s, 4H, *b*), 3.88 (s, 2H, *a*). 2.49-2.55 (m, *c* – overlaps with DMSO peak), 1.12 (t, 12H, J = 7.6 Hz, *d*). **m/z (ES$^+$-MS)** 639.3 [M+H], 661.3 [M+Na]. **Elemental analysis** calculated for $C_{35}H_{38}N_6O_6$ (%): C, 65.82; H, 6.00; N, 13.16. Found (%): C, 66.00; H, 5.89; N, 13.30.

Single Crystal X-Ray Crystallography

The X-ray single crystal data for crystals **1a** were collected using MoKα radiation (λ =0.71073Å) on a Bruker D8Venture (Photon100 CMOS detector, IμS-microsource, focusing mirrors) diffractometer equipped with a Cryostream (Oxford Cryosystems) open-flow nitrogen cryostat at the temperature 120.0(2)K. The structure was solved by direct method and refined by full-matrix least squares on F^2 for all data using Olex2 [1] and SHELXTL [2] software. All non-disordered non-hydrogen atoms were refined anisotropically, for some atoms the refinement was restrained to keep the isotropic shape of the U_{ij} components. Hydrogen atoms were placed in the calculated positions and refined in riding mode. The structure was refined as two-component twin ([-1 0 0 0 -1 0 1 0 1]; 0.863(1):0.137(1)). Crystal data and parameters of refinement are listed in Table S4. Crystallographic data for the structure have been deposited with the Cambridge Crystallographic Data Centre as supplementary publication CCDC-2124557.

Table S4. SC-XRD data for the structure of **1a**.

Empirical formula	$C_{31}H_{48}N_4O_2$
Formula weight	508.73

Temperature/K	120.0
Crystal system	monoclinic
Space group	$P2_1$
a/Å	12.3139(8)
b/Å	26.1478(16)
c/Å	39.148(2)
α/°	90
β/°	99.050
γ/°	90
Volume/Å^3	12447.9(14)
Z	16
ρ_{calc}g/cm^3	1.086
μ/mm^{-1}	0.068
F(000)	4448.0
Crystal size/mm^3	0.28 × 0.21 × 0.13
Radiation	Mo Kα (λ = 0.71073)
2Θ range for data collection/°	4.214 to 52
Index ranges	$-15 \leq h \leq 15$, $-32 \leq k \leq 32$, $-48 \leq l \leq 48$
Reflections collected	212330
Independent reflections	48818 [R_{int} = 0.1435, R_{sigma} = 0.1723]
Data/restraints/parameters	48818/127/2666
Goodness-of-fit on F^2	0.982
Final R indexes [I>=2σ (I)]	R_1 = 0.0708, wR_2 = 0.1236
Final R indexes [all data]	R_1 = 0.1685, wR_2 = 0.1534
Largest diff. peak/hole / e Å^{-3}	0.37/-0.29
Flack parameter	-2.5(5) (meaningless in the absence of heavy atoms)

1. O. V. Dolomanov, L. J. Bourhis, R. J. Gildea, J. A. K. Howard and H. Puschmann, J. *Appl. Cryst.* 2009, *42*, 339-341.

2. G.M. Sheldrick, *Acta Cryst.* 2008, *A64*, 112-122
